# Fault Diagnosis of the Autonomous Driving Perception System Based on Information Fusion

**DOI:** 10.3390/s23115110

**Published:** 2023-05-26

**Authors:** Wenkui Hou, Wanyu Li, Pengyu Li

**Affiliations:** 1 School of Reliability and Systems Engineering, Beihang University, Beijing 100191, China; zy2114120@buaa.edu.cn; 2 General Design Department, Beijing Mechanical and Electrical Engineering, Beijing 100005, China; rselipengyu@buaa.edu.cn

**Keywords:** autonomous driving, sensing system, information fusion, fault diagnosis

## Abstract

The reliability of autonomous driving sensing systems impacts the overall safety of the driving system. However, perception system fault diagnosis is currently a weak area of research, with limited attention and solutions. In this paper, we present an information-fusion-based fault-diagnosis method for autonomous driving perception systems. To begin, we built an autonomous driving simulation scenario using PreScan software, which collects information from a single millimeter wave (MMW) radar and a single camera sensor. The photos are then identified and labeled via the convolutional neural network (CNN). Then, we fused the sensory inputs from a single MMW radar sensor and a single camera sensor in space and time and mapped the MMW radar points onto the camera image to obtain the region of interest (ROI). Lastly, we developed a method to use information from a single MMW radar to aid in diagnosing defects in a single camera sensor. As the simulation results show, for missing row/column pixel failure, the deviation typically falls between 34.11% and 99.84%, with a response time of 0.02 s to 1.6 s; for pixel shift faults, the deviation range is between 0.32% and 9.92%, with a response time of 0 s to 0.16 s; for target color loss, faults have a deviation range of 0.26% to 2.88% and a response time of 0 s to 0.05 s. These results prove the technology is effective in detecting sensor faults and issuing real-time fault alerts, providing a basis for designing and developing simpler and more user-friendly autonomous driving systems. Furthermore, this method illustrates the principles and methods of information fusion between camera and MMW radar sensors, establishing the foundation for creating more complicated autonomous driving systems.

## 1. Introduction

Autonomous driving systems have emerged as a prominent development direction in the 5G era, particularly in China’s automobile industry, which increasingly focuses on intelligent vehicles [1]. However, the rise of autonomous driving has also been accompanied by notable and frequent accidents. Among the key components of autonomous driving systems, sensors, controllers, and actuators play critical roles. The sensor component, in particular, is essential for accurate positioning and obstacle detection, and a sensor failure can lead to catastrophic accidents. As a result, research on fault diagnosis of autonomous driving sensing systems has become increasingly important and is currently a topic of growing interest [2].

Autonomous vehicles necessitate the deployment of numerous sensors to gather broad information for comprehensive vehicle analysis and decision making in the face of complex road conditions [3]. With the aid of extremely effective and dependable sensor fusion algorithms, the sensors built into autonomous vehicles must deliver prompt and accurate feedback in an array of environments and usage conditions to allow continuous navigation. Due to their limited information-gathering capabilities, poor noise resistance, and low fault tolerance, the deployment of homogeneous sensors is insufficient to meet the requirements of autonomous vehicles for road condition analysis, making it impossible to achieve autonomous driving in complex situations. Research challenges for autonomous driving sensing systems currently center on two areas: (1) information fusion of data collected from multiple sensors, thereby reducing the uncertainty of information and improving the accuracy of characterization of the environment [4]; (2) choosing appropriate sensor fault diagnosis methods, thereby lowering the cost of diagnosis and reducing diagnosis time [5].

Scholars have been actively studying the first problem and exploring multi-sensor fusion algorithms that improve robustness and detection accuracy. Some scholars committed to studying multi-sensor fusion under poor conditions (weather/distance/light) have achieved significant results. For instance, Jiang et al. [6] proposed a millimeter wave (MMW) radar and camera fusion-based technique for vehicle perimeter sensing, which may improve target recognition precision in foggy circumstances. In particular, the method defogs the camera-taken images before mapping the crucial data fragments from the MMW radar to the defogged image to create the region of interest (ROI). Lastly, the detection data are combined using a weighting algorithm. Meanwhile, S. Chadwick et al. [7] developed a feature-level fusion structure to detect small objects at a distance. To extract the reflected area and velocity data from pictures and MMW radar, respectively, the structure initially employs residual networks (ResNet). Then, it stitches together radar point cloud and picture feature data. Finally, using ResNet, three-resolution feature maps are produced from the stitched features for diverse sensing applications. The proposed method balances vehicle detection accuracy and computing efficiency, achieving an optimal performance trade off. Wang et al. [8] introduced a decision-level fusion system to identify cars in wet situations. This method involves ground-calibrating the radar and camera, transmitting the radar detection results onto the camera picture to estimate target size, and leveraging the radar’s precise longitudinal distance and azimuth angle to lessen the vehicle width error introduced by image blurring. It allows for utilizing binocular vision and a traditional beam adjustment optimization synchronized point location and map-building technique. To address the challenge of real-time localization in low-light and dark environments, Zheng et al. [9] presented a novel approach based on the fusion of infrared vision and lidar for target identification and localization.

Some scholars have innovated with regard to the fusion method. Wang et al. [10] presented a synergistic fusion technique that combines a monocular camera with an MMW radar. The visual processing module processes the ROI after the MMW radar has detected the target and generated it to create a bounding box. The vehicle is then identified within the bounding box using the active contour method. Based on binocular vision sensors and the global navigation satellite system (GNSS), Yang [11] developed a multi-sensor fusion approach for attitude-tracking tasks and localization. Nabati R. [12] used the visual cone method to compare the radar detection results with the centroids of the candidate targets and then corrected the a priori detection results by regressing the candidate targets’ depth, rotation angle, and velocity. Zhou et al. [13] proposed a target-tracking technique based on multi-mode switching of onboard frequency-modulated continuous wave (FMCW) and MMW radar data fusion to improve tracking precision. They used real-world measurement data and simulated vehicle scenarios to test and validate the proposed technique, and the results demonstrate a significant improvement in tracking accuracy. Using CNNs to fuse different picture information, Zhou et al. [14] created a vehicle chassis recognition system based on multi-source sensor technologies, considerably boosting detection and classification accuracy. Meanwhile, Jia et al. [15] combined MMW radar and machine vision to detect front vehicles. To increase the system’s real-time performance and environmental flexibility, the authors used two types of cameras to capture and fuse front images: telephoto and short-focus. Additionally altered was the deep-learning algorithm’s candidate frame.

Other scholars have included drones in their research to explore new sensor-fusion techniques. Zhang et al. [16] proposed a quadrotor autonomous obstacle detection model to solve the monocular vision unmanned aerial vehicle (UAV) obstacle detection problem. The monocular estimation model offers depth information about the obstacle, whereas the target detection model delivers its position information. Hou et al. [17] developed and implemented a UAV system capable of autonomously identifying unknown external environments and automatically planning trajectories in real time to solve the problem of autonomous UAV navigation. Yang et al.’s [18] use of a multi-sensor fusion method to incorporate data from sensors such as machine vision, MMW radar, and a GPS navigation system improved the UAV’s capacity to avoid hazards such as cable poles and towers. They completed path planning and obstacle assessment using the virtual force field method (VFF).

For the second problem, some researchers use non-artificial neural network methods for sensor fault diagnosis. For example, Sharifi et al. [19] developed a probabilistic principal component analysis (MPPCA) hybrid model to identify single-sensor failure issues in nonlinear systems. Locally linear segments of the measurement space are coupled with a probabilistic principal component analysis (PPCA) model. The residual vector is built via a parity method using the transform related to each PPCA model, and Bayesian analysis is used to identify and isolate sensor failures. Meng et al. [20] suggested a method for determining whether a sensor is malfunctioning by examining the residuals between the model’s projected voltage and the sensor’s observed voltage. They used the unscented Kalman filter (UKF) algorithm to estimate the battery’s end voltage and monitor the residuals using the cumulative sum (CUSUM) approach to identify potential sensor faults based on their accumulated changes. Zhao et al. [21] introduced a time-series analysis-based fault-diagnosis method for aero-engine sensors. Their approach involves training an autoregressive moving average (ARMA) model with normal data offline and using it for real-time fault diagnosis online. Li et al. [22] offered a sensor fault-diagnosis method for acceleration sensors operating in harsh environments encountered in health monitoring systems. Their approach is based on weighted statistics of principal component analysis (PCA) residual space. Wang [23] et al. presented a generation approach based on integrating information-geometry causal inference (IGCS) and K2 score search strategy to improve the hydraulic condition monitoring system’s problem identification precision.

Others use artificial neural network methods for sensor fault diagnosis. Mariam et al. [24] used automated associative neural networks (AANN) to identify single and many sensor failures in a system. The model could validate sensor measurements via sensor error correction, missing data substitution, and noise filtering. Guo et al. [25] proposed a hybrid feature model combined with deep learning to identify UAV sensor flaws. The method utilizes the short-time Fourier transform (STFT) to transform residual signals of various sensor faults into corresponding time–frequency maps. The features of the images are then recovered using a convolutional neural network (CNN). Zhang et al. [26] developed a low-power multi-sensor vibration signal fault diagnostic technique (MLPC-CNN). To accurately extract grayscale image properties from multi-sensor data, they introduced a single sensor to single channel convolution (STSSC) approach. Next, use a mean pool layer-based bypass branching structure to preserve low-dimensional data while extracting high-level characteristics. Then, adding a multilayer pool classifier decreases the number of network parameters and removes the possibility of overfitting. Li et al. [27] collected time and frequency domain features and morphological information from multi-dimensional aero-engine sensor signals to represent the sensor’s health status and suggested an improved Henry gas solubility optimization approach for feature selection. Ultimately, they used the feature vector as the sensor’s health indicator to perform intelligent defect diagnoses using deep belief networks (DBN). Guo et al. [28] proposed a structural acceleration sensor fault self-diagnosis and fault signal self-recovery algorithm, combining CNN and deep convolutional generative adversarial networks (DCGAN). Zhang et al. [29] suggested a CGA-LSTM-based sensor failure detection technique that first used CNN to extract features from data, then integrated with the Long Short-Term Memory (LSTM) model, and last employed a Genetic algorithm (GA) to optimize the essential hyper-parameters in the LSTM network. Ma et al. [30] developed a fault-detection technique for multi-source sensors that can diagnose fixed deviation and drift deviation faults in complex systems. This method employs a CNN to extract features between different sensors and recurrent networks to describe the temporal properties of the sensors. Lin [31] suggested a hybrid approach for sensor fault diagnosis and fault data reconstruction based on enhanced LSTM and random forest (RF).

In conclusion, this research contributes in the following two ways:

(1) We propose a space–time fusion algorithm to combine data from a single MMW radar with a single camera sensor;
(2) To determine the effect of each failure mode on the sensor, we developed an information-fusion-based fault diagnosis method.

The rest of this paper is structured as follows: Section 2 briefly introduces the theory. Section 3 explains the proposed information fusion and fault diagnosis method. Section 4 shows the simulation process and the experimental results. Finally, Section 5 offers the conclusion.

## 2. The Theory

### 2.1. The Failure Criteria of the Autonomous Driving Perception System

Fault standards for autonomous driving perception systems mainly involve fault detection and diagnosis, and their standards are usually formulated based on system performance and reliability requirements. Failure criteria usually include the following aspects [32].

- Fault classification: Classify possible faults for better fault diagnosis and repair.
- Fault detection accuracy: Require and evaluate the accuracy of fault detection to ensure that the detected faults actually exist.
- Fault diagnosis accuracy: Require and evaluate the accuracy of fault diagnosis to ensure that the determined cause and location of the fault are correct.
- Fault response time: Require and evaluate the response time of fault repair to ensure timely repair when a fault occurs.
- Fault tolerance: Require and evaluate the system’s tolerance to different types of faults to ensure that the system can still operate normally when some faults occur.

In this paper, we evaluate the performance and reliability of the autonomous driving perception system by analyzing the fault classification threshold, fault diagnosis accuracy, and time to fault detection.

The performance evaluation of automotive data fusion often requires summarizing aspects of perceptual performance into a small number of scalar values for comparison [33]. Computing lower-level metrics requires associating the estimated tracks of the System-Under-Test to their corresponding reference tracks, which is realized in the following way: the pairwise distances between all estimated and all reference objects or tracks are computed using an object distance function [34,35]. In this paper, we use the Euclidean distance τ of the observation point of the MMW radar and the center point of the camera sensor to associate them and then use the ratio of τ to the vehicle length W as the threshold for fault classification. The specific operation is given in Section 3.3.

### 2.2. Information Fusion

The general definition of information fusion can be roughly summarized as follows: information fusion is the study of efficient methods for automatically or semi-automatically transforming information from different sources and different points in time into a representation that provides effective support for human or automated decision making [36,37]. The traditional multi-source information fusion theory includes data-level information fusion, feature-level information fusion, and target-level data fusion [4].

**Figure 1:**
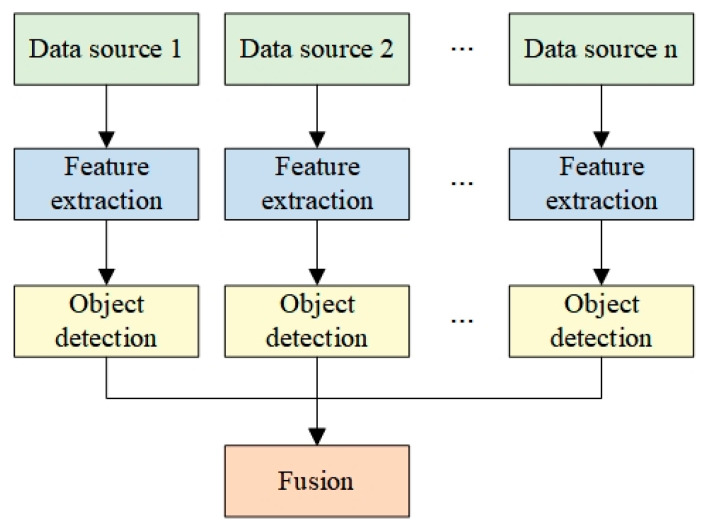
Target-level data fusion.

This paper uses target-level data fusion, as shown in Figure 1: we perform feature extraction on the data from different sensors, process them according to the requirements of object detection, and ultimately output the discrimination result. Target-level data fusion is more fault-tolerant and real-time [38].

### 2.3. Caffe-Based CNN

A CNN is a multilayer network structure designed for image-recognition tasks. The network employs unsupervised learning for training and includes an input layer, an output layer, a convolutional layer, and a pooling layer [39]. The convolutional layer primarily utilizes a sampler to capture essential data content from the input data. The pooling layer’s objective is to reduce the size of the feature map, control overfitting, and shorten the training time by limiting the number of parameters and computations [40]. The fully connected layer transforms the two-dimensional feature map from the convolution output into a one-dimensional vector, then transmits the output values to the classifier for mapping to the sample label space [41]. To overcome the redundant parameters of the fully connected layer, high-performing network models such as ResNet and GoogLeNet employ global average pooling instead, with loss functions such as softmax as the network objective function [42,43], which fuses the learned deep features.

**Figure 2:**
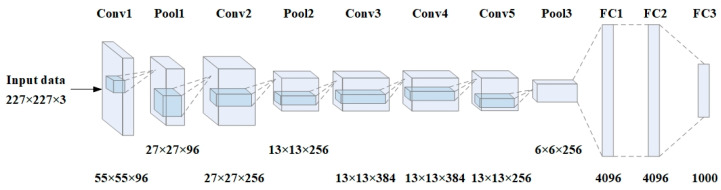
AlexNet network structure.

We selected the AlexNet network as the model for Caffe. It consists of eight layers, including five convolutional layers and three fully connected layers. Maximum pooling is applied after the first, second, and fifth convolutional layers. Figure 2 illustrates the network structure and output feature map size of each layer in the network.

### 2.4. Coordinate Calibration

The purpose of the coordinate calibration is to obtain the absolute position of the target [44]. The following describes the three coordinate systems involved in this paper, as well as the rotation matrix used to represent the relative direction between two space coordinates.

#### 2.4.1. Coordinate System

1. 1. Image coordinate system

**Figure 3:**
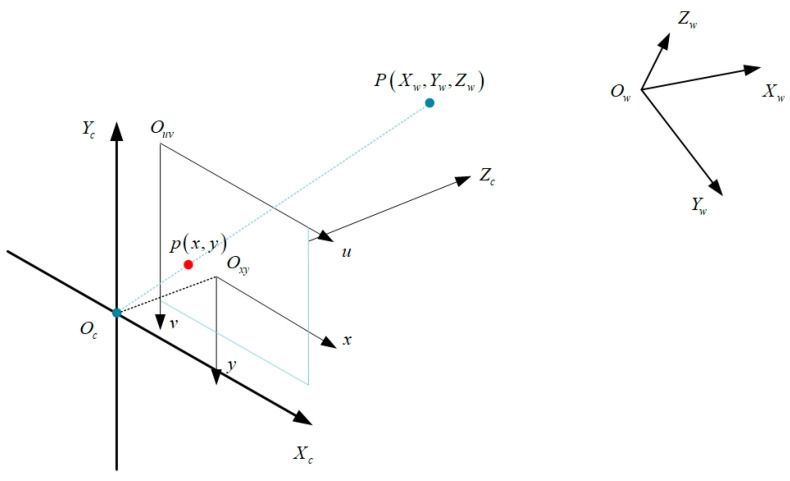
Image coordinate systems, camera coordinate systems, and world coordinate systems.

There are two different kinds of coordinate systems for images: pixel coordinate systems and physical coordinate systems [45]. The original point of the pixel coordinate system is in the upper left corner of the image, as shown in the $O_{uv}$ of Figure 3, which represents the logical distance between targets in the picture; the original point of the physical coordinate system is in the center of the image, as shown in the $O_{xy}$ of Figure 3, which represents the real distance between targets in space.

Following are the steps involved in converting a physical coordinate system $O_{xy}$ to a pixel coordinate system $O_{uv}$.

(1) $$\left[\begin{array}{l} u\\ v\\ 1 \end{array}\right]=\left[\begin{array}{ccc} 1/dx & 0 & u_{0}\\ 0 & 1/dy & v_{0}\\ 0 & 0 & 1 \end{array}\right]\left[\begin{array}{l} x\\ y\\ 1 \end{array}\right]$$

2. Camera coordinate system

The camera coordinate system is a three-dimensional spatial coordinate system, and the object on this coordinate system is inverted on the image coordinate system [46]. As illustrated in Figure 3, $O_{c}$ is the camera optical center; the $Z_{c}$ axis along the camera optical axis and the image plane is perpendicular to the direction of the image and is positive; the $X_{c},Y_{c}$ axis parallels the image physical coordinate system x,y axis; and $O_{c},\;O_{xy}$ is its focal length f. $O_{c}X_{c}Y_{c}Z_{c}$ forms the camera coordinate system.

Following are the steps involved in converting a camera coordinate system $O_{c}$ to a physical image coordinate system $\;O_{xy}$.

(2) $$Z_{c}\left[\begin{array}{l} x\\ y\\ 1 \end{array}\right]=\left[\begin{array}{cccc} f & 0 & 0 & 0\\ 0 & f & 0 & 0\\ 0 & 0 & 1 & 0 \end{array}\right]\left[\begin{array}{l} X_{c}\\ Y_{c}\\ Z_{c}\\ 1 \end{array}\right]$$

3. World coordinate system

The camera’s position and any other object in the environment relationship can be represented using the world coordinate system. Figure 3 depicts the link between the world coordinate system and various coordinate systems.

Following are the steps involved in converting a world coordinate system $O_{w}$ to a camera coordinate system $O_{c}$.

(3) $$\left[\begin{array}{l} X_{c}\\ Y_{c}\\ Z_{c}\\ 1 \end{array}\right]=\left[\begin{array}{cc} R & T\\ \vec{0} & 1 \end{array}\right]\left[\begin{array}{l} X_{w}\\ Y_{w}\\ Z_{w}\\ 1 \end{array}\right]$$

In the above equation, R is a 3 × 3 orthogonal unit matrix (also called a rotation matrix), and T is a three-dimensional translation vector. The vector $\vec{0}=\left(0,0,0\right)$.

#### 2.4.2. Rotation Matrix

Two coordinate systems with the same origin can be rotated around their axes using a rotation matrix [47,48]. In a right-handed coordinate system, if the angles x, y, and z are rotated around the ψ, φ, and θ axes in turn, then the total rotation matrix $R=R_{x}\left(\psi \right)\cdot R_{y}\left(\varphi \right)\cdot R_{z}\left(\theta \right)$.

The active rotation around the x-axis (counterclockwise in the *y*–*z* plane) is defined as

(4) $$R_{x}\left(\psi \right)=\left[\begin{array}{ccc} 1 & 0 & 0\\ 0 & \cos\psi & -\sin\psi \\ 0 & \sin\psi & \cos\psi \end{array}\right].$$

The active rotation around the y-axis (counterclockwise in the *x*–*z* plane) is defined as

(5) $$R_{y}\left(\varphi \right)=\left[\begin{array}{ccc} \cos\varphi & 0 & \sin\varphi \\ 0 & 1 & 0\\ -\sin\varphi & 0 & \cos\varphi \end{array}\right].$$

The active rotation around the z-axis (counterclockwise in the *x*–*y* plane) is defined as

(6) $$R_{z}\left(\theta \right)=\left[\begin{array}{ccc} \cos\theta & -\sin\theta & 0\\ \sin\theta & \cos\theta & 0\\ 0 & 0 & 1 \end{array}\right].$$

## 3. The Proposed Method

The key issues to be addressed in this study are as follows:

(1) Realize the information fusion of a single camera and a single MMW radar sensor;
(2) Use information fusion from multiple sensors to diagnose sensor failures and evaluate the accuracy of fault diagnosis and its impact on autonomous driving safety.

**Figure 4:**
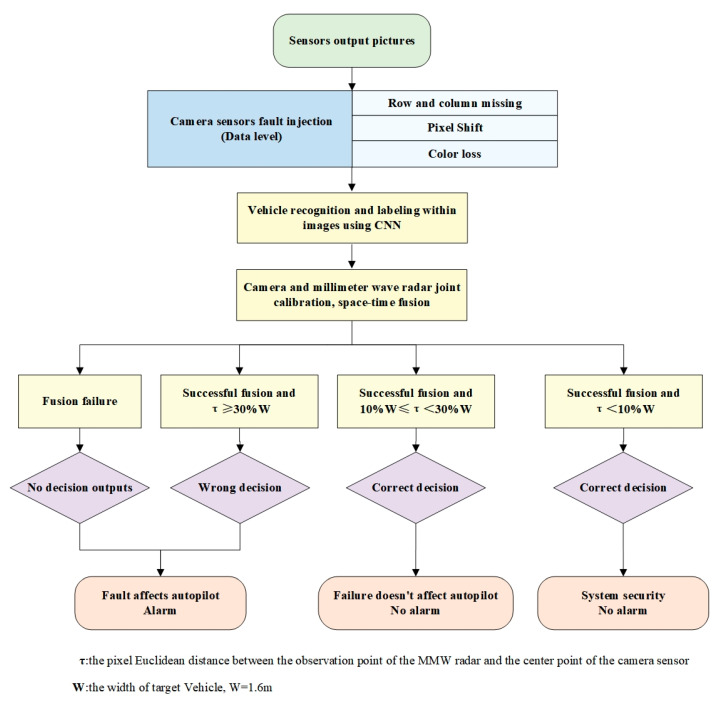
Flowchart of fault diagnosis based on information fusion.

As illustrated in Figure 4, the fault diagnosis procedure described in this research uses three major methods: a fault diagnostic method to pinpoint camera sensor failures, an information fusion algorithm to achieve the space–time fusion of two sensors, and CNN for vehicle recognition and labeling.

The fault diagnosis process of the perception system proposed in this paper is as follows:

(1) Perform Prescan/Matlab co-simulation to obtain data output of a single camera sensor and a single MMW radar sensor;
(2) Define and simulate the sensor failure. The MMW radar simulation provides a direct output of the target point and has a single failure mode. Therefore, we assumed that there were no failures. Only three failure modes are injected into the camera sensor;
(3) Use the Caffe-based CNN (AlexNet) to identify and label vehicles in the images obtained in the first step;
(4) Carry out joint calibration and data fusion of the MMW radar sensor and the camera sensor;
(5) Study the fusion results under different failure modes;
  - The index: τ—the pixel Euclidean distance between the observation point of the MMW radar and the center point of the camera sensor; W—the width of the target vehicle, W = 1.6 m.
  - The strategy: if fusion fails, or τ≥30% W, it will be regarded as a camera sensor failure, and an alarm will be issued; if the fusion succeeds and 10% W≤τ<30% W, it will still be regarded as a camera sensor failure, but no alarm will be issued, and the system will only reduce the level of automatic driving; if the fusion succeeds and τ<10% W, it will be regarded as a fusion error, with no alarm, and the system is safe.
(6) Compare the fusion result with the threshold and draw a conclusion.

### 3.1. CNN-Based Vehicle Recognition Algorithm

Although R-CNN, Faster R-CNN, and YOLO perform well in detection accuracy, their training processes are multi-stage, and the training cost is relatively expensive in time and space [49,50,51]. This paper focuses on injecting faults into sensor output images and exploring the correlation between information fusion and fault diagnosis based on the diagnostic results, as well as their impact on autonomous driving safety. Additionally, the computational resources in our laboratory are limited. Therefore, we ultimately chose a Caffe-based CNN (AlexNet) with only a one-stage training process to explore a target detection method that saves on training costs.

#### 3.1.1. Image Preprocessing

The data obtained by the camera are in the form of RGB images, which have a high pixel count that can adversely impact real-time processing. To facilitate image recognition, we converted these images into grayscale [52]. Gray image conversion is usually performed by weighting the three primary colors of red, green, and blue with a coefficient of 0.3, 0.59, and 0.11 [53]. These coefficients are determined based on the human luminance perception system’s adjustment.

(7) VGary=0.3×VRed+0.59×VGreen+0.11×VBlue

#### 3.1.2. CNN Based on the Caffe Framework

**Figure 5:**
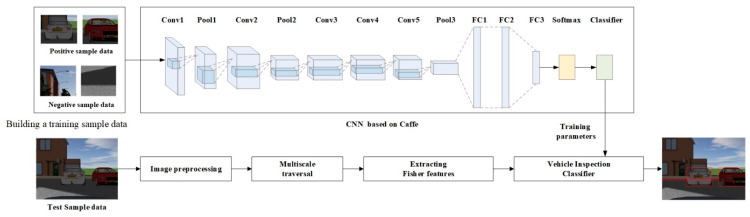
The CNN-based research framework for vehicle recognition.

In this study, we added a classifier for vehicle target detection at the end of the neural network, used the AlxeNet network to perform image recognition and classification on the images obtained by Prescan simulation, and then used the trained classifier to detect vehicle targets. Most of the images generated by the PreScan simulation are used as our training set (“positive samples” containing vehicle images, “negative samples” not containing vehicle images), and the remaining images constitute the test set. After multi-scale training and Fisher feature extraction, we input the test set to the trained vehicle detection classifier for vehicle classification and labeling. The concept of this study is shown in Figure 5.

### 3.2. Information Fusion Algorithm Based on Joint Sensor Calibration

The purpose of this research is to investigate the information fusion method of a single camera and a single MMW radar in autonomous driving. This approach was chosen for several reasons:

- Cameras and MMW wave radars are well-established sensing techniques in the field of autonomous driving, with proven success in detecting objects and providing valuable information about the environment [54,55];
- The camera provides high-resolution texture information of the surrounding environment of the vehicle and exhibits excellent performance in terms of horizontal position, horizontal detection distance, and target classification ability, but the camera performs poorly in terms of the vertical detection distance [12]. The MMW radar is highly robust against various lighting conditions and is less susceptible to adverse weather conditions such as fog and rain compared to cameras [4]. However, classifying objects by radar is very challenging due to the low resolution of radar [56];
- Integrating data from different sensors can significantly enhance the accuracy and reliability of autonomous driving systems [57,58].

In summary, the use of cameras and MMW radars for information fusion has great potential to enhance the accuracy and reliability of autonomous driving systems, making them an important focus of this study.

Although multiple cameras and MMW radar sensors are commonly used in self-driving cars, investigating autonomous driving technologies that utilize only a single camera and a single MMW radar to fuse information remains of practical interest. This approach can potentially reduce the cost of autonomous driving systems by decreasing the number of sensors, which can lower manufacturing costs and energy consumption. Furthermore, this approach can facilitate the development of simpler and more straightforward autonomous driving systems. Additionally, this approach can offer insight into the principles and methods of information fusion between cameras and MMW radar sensors, serving as a foundation for developing more complex autonomous driving systems.

#### 3.2.1. MMW Radar, Camera Model, and Coordinate Selection

**Figure 6:**
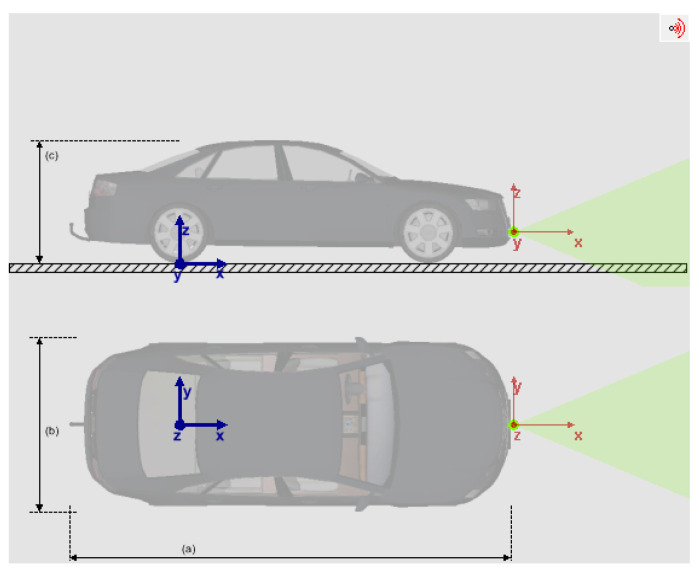
Radar model installation position. (**a**) The length of the vehicle. (**b**) The width of the vehicle. (**c**) The height of the vehicle.

The origin of the vehicle coordinate system, denoted by the letters $O_{W}X_{W}Y_{W}Z_{W}$ in this paper, is the central position of the rear axle at zero height above the ground. As seen in Figure 6, the vehicle coordinate system, which is blue, complies with the right-hand rule.

We utilized the ARS408 MMW radar, which has an actual refresh rate of approximately 20 Hz, a frame rate of 15 FPS, and a cycle period of roughly 67 ms. Figure 6 displays the MMW radar coordinate system in the green area, where the coordinate origin is at the center of the vehicle’s front bumper, and the coordinate plane is parallel to the vehicle’s coordinate plane.

**Figure 7:**
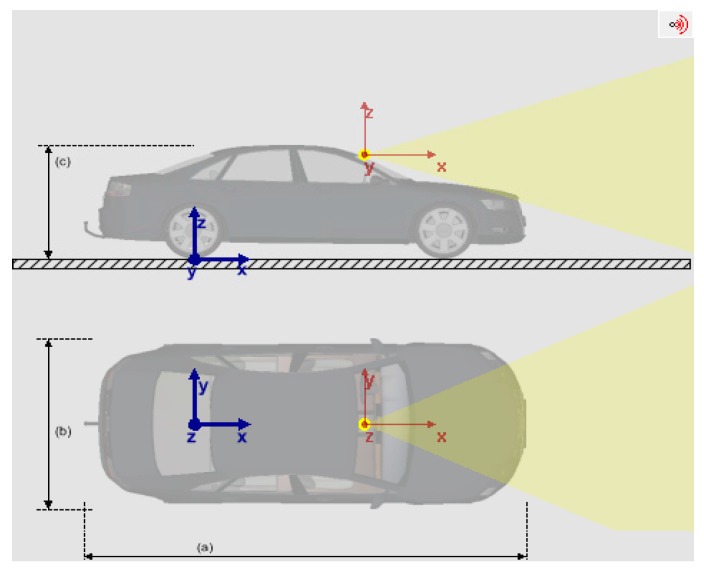
Camera model installation position. (**a**) The length of the vehicle. (**b**) The width of the vehicle. (**c**) The height of the vehicle.

Additionally, we employed the LI-USB30-AR023ZWDR camera, which has an actual refresh rate of 20 Hz, a frame rate of 20 FPS, and a cycle time of 50 ms. Figure 7 shows the camera coordinate system in the yellow area, with the coordinate origin behind the front windshield and the coordinate plane parallel to the vehicle’s coordinate plane.

#### 3.2.2. Joint MMW Radar and Camera Calibration

The distance from the target item to the radar panel, the angle between the line connecting the target object to the radar panel and the normal radar panel, and the relative velocity to the radar are all output by conventional MMW radar, which can identify up to 64 detection targets. The output angle value is positive if the item is on the right side of the radar; otherwise, it is negative. In this study, we state that an MMW radar can produce up to 20 targets, and we represent the MMW radar’s three-dimensional coordinates as $O_{r}(R_{r},\theta_{r},V_{r})$, with the radar panel’s center point serving as the origin.

Since the MMW radar detection sweep plane is two-dimensional, this paper assumes that the radar plane normally is parallel to the ground and does not output z-axis direction information. The following is an expression for the conversion relationship between the world coordinate system $O_{W}$ and the radar coordinate system $O_{R}$:

(8) $$\left(\begin{array}{l} x_{r}\\ y_{r}\\ z_{r}\\ 1 \end{array}\right)=\left(\begin{array}{cc} R_{z} & T\\ \vec{0} & 1 \end{array}\right)\left(\begin{array}{l} x_{w}\\ y_{w}\\ z_{w}\\ 1 \end{array}\right).$$

We can substitute into Equation (6) and simplify as follows:

(9) $$\left(\begin{array}{l} x_{r}\\ y_{r}\\ 1 \end{array}\right)=\left(\begin{array}{ccc} \cos\theta & -\sin\theta & x_{0}\\ \sin\theta & \cos\theta & y_{0}\\ 0 & 0 & 1 \end{array}\right)\left(\begin{array}{l} x_{w}\\ y_{w}\\ 1 \end{array}\right).$$

θ represents the angle between the radar coordinate system and the world coordinate system, and $x_{0},y_{0}$ can be solved by selecting two sets of characteristic points and bringing them into Equation (10).

The conversion from the radar coordinate system $O_{R}$ to the camera coordinate system $O_{C}$ is as follows.

(10) $$\left(\begin{array}{cc} R_{r}^{c} & t_{r}^{c}\\ 0 & 1 \end{array}\right)=\left(\begin{array}{cc} R_{w}^{c} & t_{w}^{c}\\ 0 & 1 \end{array}\right)\left(\begin{array}{cc} R_{w}^{r} & t_{w}^{r}\\ 0 & 1 \end{array}\right).$$

$R_{w}^{c}$ and $t_{w}^{c}$ denote the conversion matrix from the radar coordinate system to the camera coordinate system, and $R_{w}^{r}$ and $t_{w}^{r}$ denote the conversion matrix from the radar coordinate system to the world coordinate system.

#### 3.2.3. Space–Time Fusion of MMW Radar and Camera

First, the spatial integration of the camera and MMW radar is accomplished. The MMW radar detection sweep plane is two-dimensional, so it is possible to think of the transformation of the radar coordinate system $O_{R}$ to the camera coordinate system $O_{C}$ as the transformation of the two-dimensional coordinate system. The procedure for converting radar data $\left(x_{r},y_{r}\right)$ to image coordinates $\left(u,v\right)$ can be easily determined by consulting the literature [59].

(11) $$\left(\begin{array}{l} u\\ v\\ 1 \end{array}\right)=T_{I}^{R}\left(\begin{array}{l} x_{r}\\ y_{r}\\ 1 \end{array}\right)=\left(\begin{array}{ccc} t_{11} & t_{12} & t_{13}\\ t_{21} & t_{22} & t_{23}\\ t_{31} & t_{32} & t_{33} \end{array}\right)\left(\begin{array}{l} x_{r}\\ y_{r}\\ 1 \end{array}\right)$$

By replacing n joint calibration points (where *n* < 3), the matrix least-squares method can be used to generate the coefficients for the transformation matrix $T_{I}^{R}$ to be solved. Assuming that $\left(x_{r}^{j},y_{r}^{j}\right)$ and $\left(u_{j},v_{j}\right)$ are the jth pair of calibration points, the pending parameters can be written as follows [6,59]:

(12) $$T_{I}^{R}={\left(\left(\begin{array}{ccc} x_{r}^{1} & \cdots & x_{r}^{n}\\ y_{r}^{1} & \cdots & y_{r}^{1}\\ 1 & \cdots & 1 \end{array}\right){\left(\begin{array}{ccc} x_{r}^{1} & \cdots & x_{r}^{n}\\ y_{r}^{1} & \cdots & y_{r}^{1}\\ 1 & \cdots & 1 \end{array}\right)}^{T}\right)}^{-1}\left(\begin{array}{ccc} x_{r}^{1} & \cdots & x_{r}^{n}\\ y_{r}^{1} & \cdots & y_{r}^{1}\\ 1 & \cdots & 1 \end{array}\right){\left(\begin{array}{ccc} u_{1} & \cdots & u_{n}\\ v_{1} & \cdots & v_{n}\\ 1 & \cdots & 1 \end{array}\right)}^{T}.$$

**Figure 8:**
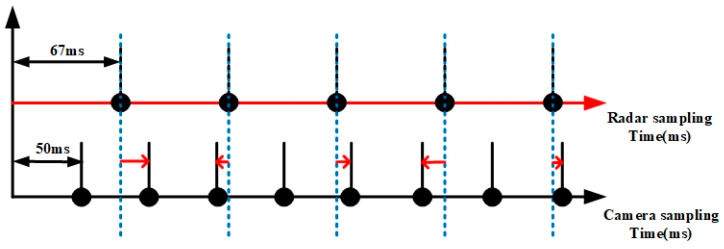
Temporal fusion between MMW radar and camera.

The MMW radar and camera, which are fused in time, come next. For this, data collected from the radar and camera must be coordinated. Because the frame rates of the radar and camera employed in this study are around 15 FPS and 20 FPS, respectively, we use the measurement time of the lower-frequency MMW radar as a benchmark to make the higher-frequency camera data backward-compatible. Since there is no variation among the images the camera took throughout its 50 ms acquisition period, we may instead utilize the image closest to the reference time to calibrate the MMW radar camera’s timing. This is displayed in Figure 8.

### 3.3. Fault Diagnosis Method Based on Information Fusion

Since there is only one failure mode for MMW radar, this research exclusively considers the failure mode of the camera sensor. To verify the suggested strategy, three camera sensor failure scenarios and one typical instance are defined, as demonstrated in Table 1 below.

In this study, the LI-USB30-AR023ZWDR camera had a frame rate of 30 FPS, allowing the rendering of 30 images or frames per second. The cycle period of this camera is 50 ms, representing the time between two consecutive photographs. In contrast, the ARS408 MMW radar’s frame rate is approximately 15 FPS, enabling it to process 15 images per second. This radar’s cycle period is 67 ms, denoting the interval between two successive images. Hence, it is apparent that there exists an approximate 25% difference between the camera and MMW radar’s processing time.

We have increased the range of permissible errors to consider the many operational situations that cameras and MMW radars may experience, including changes in lighting and unfavorable weather such as rain and snow. Specifically, we have defined the threshold for “failure” as a deviation equal to or greater than 30%, while the threshold for “no failure” is less than 10%. Deviations that fall between these two thresholds are categorized as “deviation failure”.

**Figure 9:**
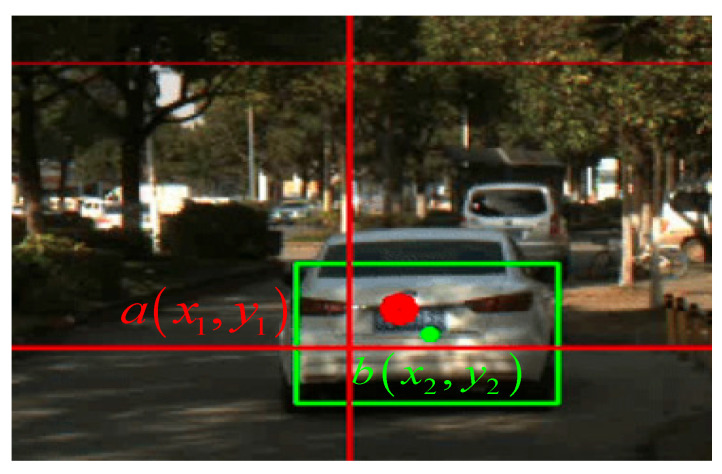
Two types of sensors labeled with center points.

The following steps are used in the fault diagnosis based on the information fusion process: first, establish a pixel coordinate system to determine the Euclidean distance τ between observation point $b\left(x_{2},y_{2}\right)$ of MMW radar and the centroid $a\left(x_{1},y_{1}\right)$ of visual markers [33]. The centers of the two sensors are seen in Figure 9.

(13) $$\tau =d_{ab}=\sqrt{{\left(x_{1}-x_{2}\right)}^{2}+{\left(y_{1}-y_{2}\right)}^{2}}$$

Then, comparing the pixel distance with the target width W of the body (the target width W is estimated to be 1.6 m based on engineering experience), the problem diagnostic process is illustrated in Figure 4.

Since the vision sensor output is set to 320 × 240 in the PreScan autonomous driving scene modeling, the pixel width/physical width should be adjusted to 1:64.

## 4. Simulations Experiments and Results Analysis

**Figure 10:**
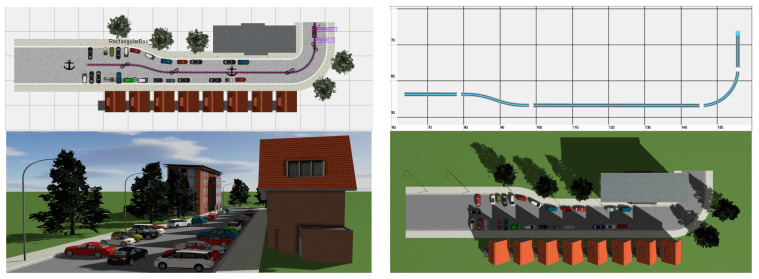
The simulation environment.

As illustrated in Figure 10, a simulation environment is designed for this paper using Prescan8.5.0/ MATLAB R2014a. The primary components are a driving vehicle module with a control system, straight and curving road conditions, roadside obstacle vehicles, buildings and trees, and weather lighting. All of the vehicles, with the exception of the primary vehicle, are parked on the side of the road and move at a speed of 5 m/s.

The motion parameters and the settings for the two sensors are set as shown in the following Table 2 and Table 3.

**Figure 11:**
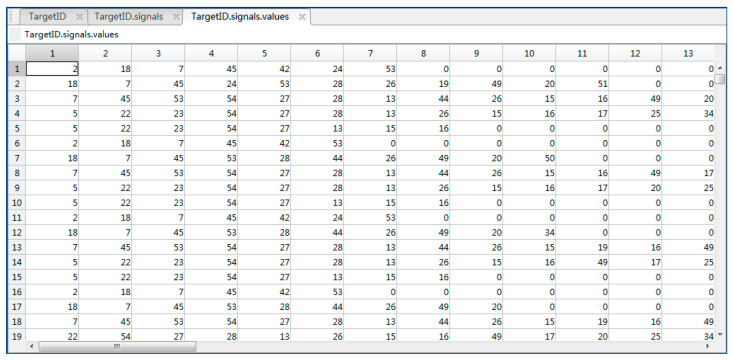
Numerical matrix of the MMW radar.

Accounting for the potential interference of weather on the output of MMW radar, we introduced Gaussian noise to the radar signal in the simulation environment. The chosen MMW radar sensor can directly output the target information, the frequency is 20 Hz, and the experiment time is 8 s. Figure 11 displays the 160 groups of detection results.

**Figure 12:**
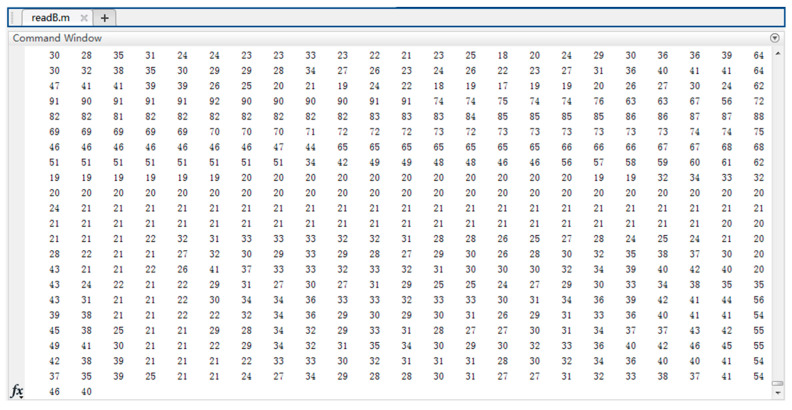
Numerical matrix of the camera output layer (B).

**Figure 13:**
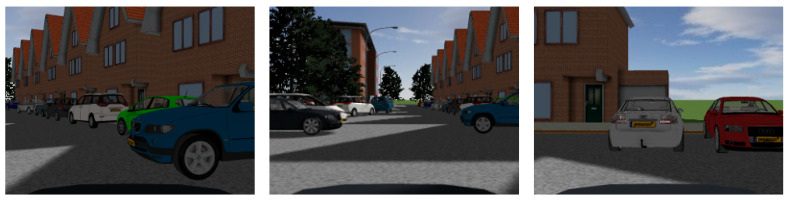
RGB images converted from camera numerical matrix.

To preserve more information about targets, we configure the camera sensor to output a color image. The output data has time-series characteristics and is displayed as numerical matrixes with three levels, R, G, and B, as illustrated in Figure 12. Using the in-built conversion module of Matlab, we transform the numerical matrix generated by the camera sensor into an RGB image, as shown in Figure 13.

### 4.1. Vehicle Detection Based on Vision Sensors

**Figure 14:**
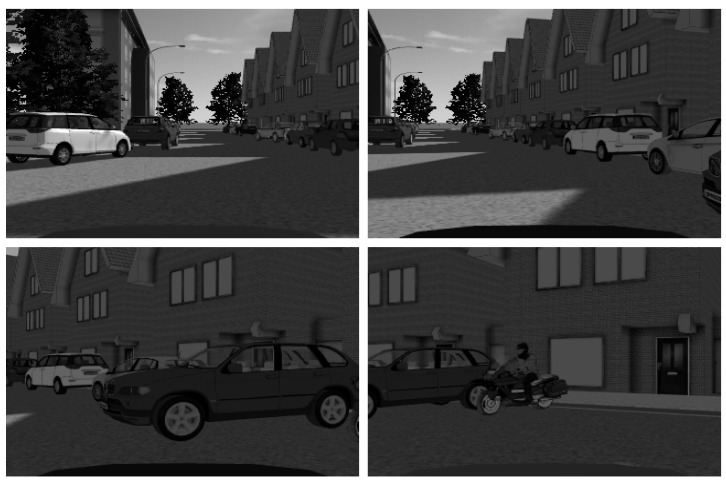
Sensor output results after grayscale processing.

According to the method in Section 3.1.1, the RGB images converted from the camera’s numerical matrix are grayed out, and the results are shown in Figure 14.

**Figure 15:**
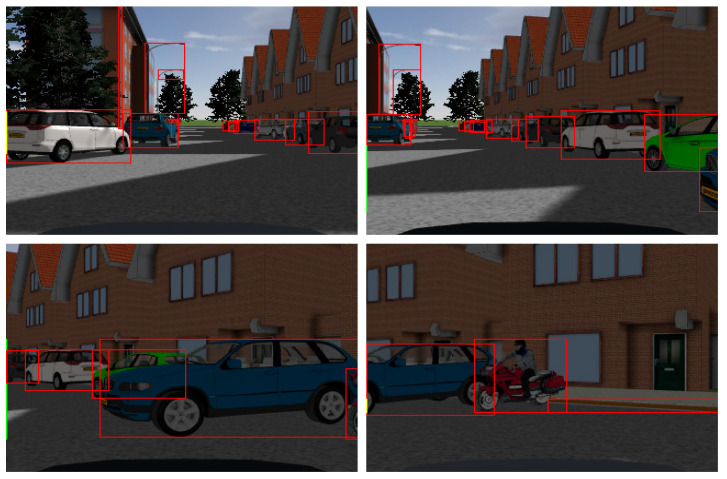
CNN identifies and marks vehicles. The red boxes indicate the objects marked by CNN.

Figure 15 below displays the outcomes of utilizing a CNN to identify and label the vehicles in the photos.

### 4.2. Sensor Information Fusion Based on Joint Calibration

Target detection is carried out on this ROI by projecting the target point received by the MMW radar sensor onto the camera image and incorporating a priori knowledge. The exact procedure is as follows: import the output from the MMW radar and camera sensors, dynamically alter the external parameters, and assume this parameter is right if the rectangular box on the image is in the intended location. By fixing the external parameters, sensor information fusion based on joint calibration can be performed.

**Figure 16:**
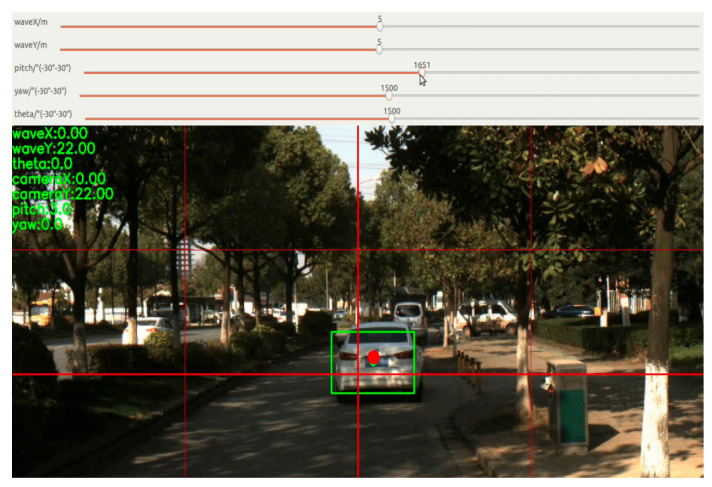
Program running interface.

**Figure 17:**
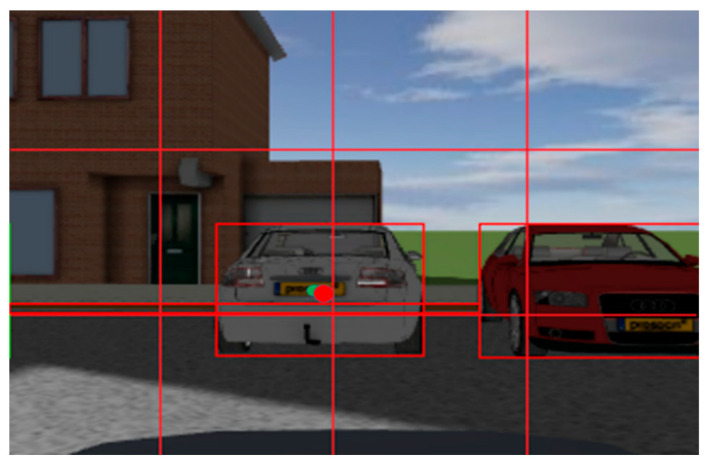
Joint calibration result. The red boxes indicate the vehicles marked by CNN. The green circle indicates the millimeter-wave radar observation point (default at the center of the vehicle). The red circle indicates the center point of the camera annotation (the center point of the visual annotation).

The output from the MMW radar and camera sensors appears in Figure 16 below, and the joint calibration is completed by modifying the external parameter (i.e., Euler angle), according to Figure 17, where the red dot denotes the center of the vehicle image labeling, and the green dot denotes the target point of radar recognition (default at the center of the vehicle).

### 4.3. Fault Simulation and Fault Identification Method Examination

Corresponding to Section 3.3, the failure modes of the vision sensor studied in this paper include missing row/column pixels, pixel displacement, and target color loss. The objective of this experiment is to independently evaluate the impact of each failure mode on image quality by administering them individually. Concurrent injection of multiple fault modes can interfere or overlap, posing challenges to determining the isolated contribution of each failure mode.

#### 4.3.1. Missing Row/Column Pixel Failure

**Figure 18:**
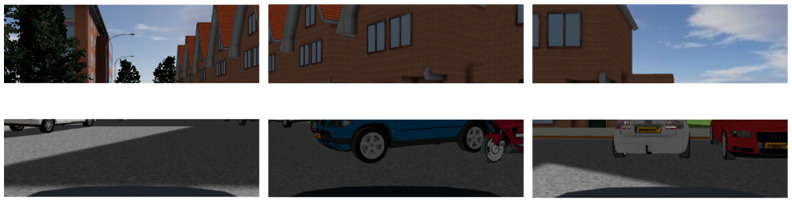
Missing row/column pixel fault characterization.

It is shown that some or all of the rows/columns are missing pixels, and the output data are missing and replaced by white pixel blocks in the image. The output image is 320 × 240 pixels so that 100 to 140 horizontal rows become white pixel blocks, as shown in Figure 18.

After taking ten photos from each of the three sets of fault samples, Table 4 displays the resulting Euclidean distance.

**Figure 19:**
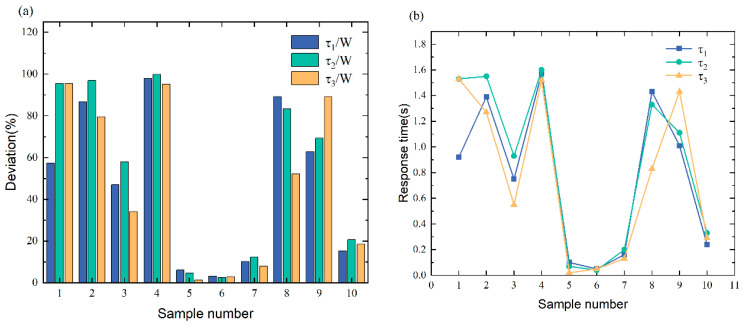
(**a**) The resulting statistics of missing row/column pixel fault identification. (**b**) The curve of missing row/column pixel fault response time.

Figure 19 shows that the $\tau /W\left(\%\right)$ typically falls between 34.11% and 99.84%, with a response time of 0.02 s to 1.6 s. According to these results, the deviations are between 30% and 100%, which means that our method can effectively and promptly identify the missing row/column pixel failure. However, some of the data fall within the [10,30] interval, contrary to what is expected. Because the fault injection is not ideal, the target vehicle is not adequately covered by the white pixel patches in these data images. The unexpected results indicate that the failure of missing row/column pixels can greatly compromise the safety of autonomous vehicles. This can lead to inaccurate or unrecognizable target recognition, prompting an alarm, reducing the autonomy level, or requiring the vehicle to stop.

#### 4.3.2. Pixel Shift Fault

**Figure 20:**
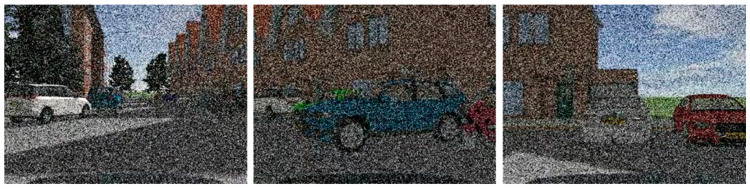
Pixel displacement fault characterization.

This issue is manifested as a blurred or distorted image, as shown in Figure 20.

Ten photos from each of the three sets of fault samples were taken, and Table 5 displays the resulting Euclidean distance.

**Figure 21:**
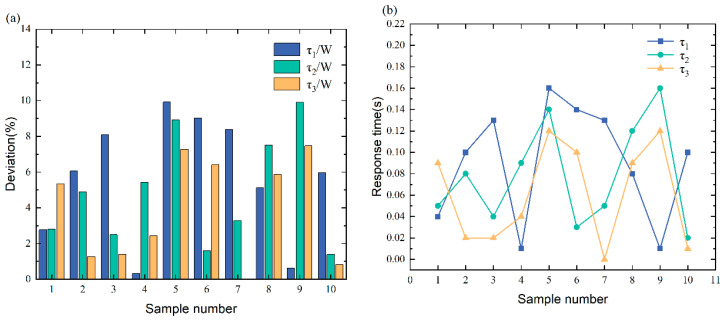
(**a**) The resulting statistics of pixel displacement fault identification. (**b**) The curve of pixel displacement fault response time.

Figure 21 shows that the $\tau /W\left(\%\right)$ typically falls between 0.32% and 9.92%, with a response time of 0 s to 0.16 s. Based on the results, the deviations are between 0 and 10%, which deviates from the prediction. This is because this pixel shift fault is reproduced by increasing the target vehicle’s pixel noise without altering the target vehicle’s appearance or color, which does not influence picture identification. Hence, when such faults occur, the sensor data fusion remains stable to ensure safe autonomous driving, and the system function remains operational.

#### 4.3.3. Target Color Loss Fault

**Figure 22:**
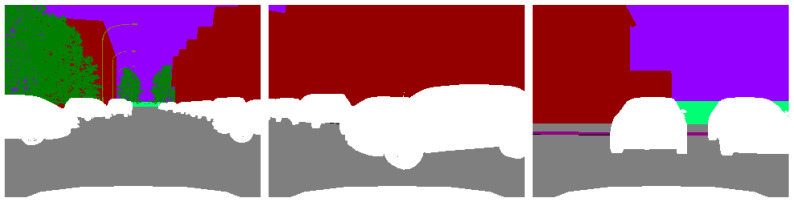
Target color loss fault characterization.

The performance is a complete loss or assimilation of the color of a class of targets, with the actual output of part of the pixel block data being the same, as shown in Figure 22. The different color indicates objects color loss.

Ten photos from each of the three groups of fault samples were collected, and Table 6 displays the resulting Euclidean distance.

**Figure 23:**
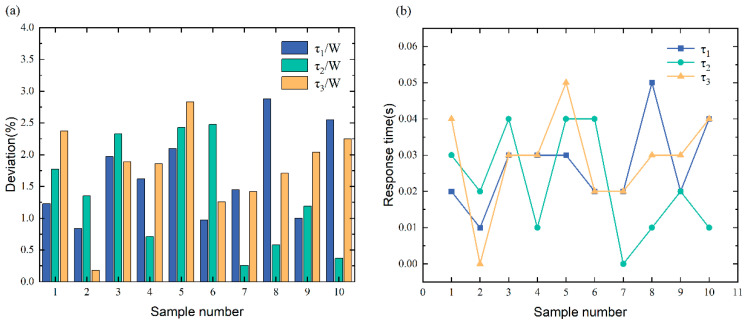
(**a**) The resulting statistics of target color loss fault identification. (**b**) The curve of target color loss fault response time.

As seen in Figure 23, the $\tau /W\left(\%\right)$ typically falls between 0.26% to 2.88%, with a response time of 0 s to 0.05 s. Based on the results, the deviations are between 0 and 3%, which is unexpected. This is because this fault is reproduced by changing the pixel color of the target vehicle without altering the target vehicle’s appearance, which does not influence picture identification. Hence, when such faults occur, the sensor data fusion remains stable to ensure safe autonomous driving, and the system function remains operational.

## 5. Conclusions

Based on the information fusion of the MMW radar and the camera, this research offers a fault diagnosis method for an autonomous driving sensing system. First, we generated an autonomous driving simulation scenario using PreScan and collected sensor data from a single MMW radar and a single camera. Then, we identified and labeled the automobiles in the photos using a CNN. Third, we suggested a sensor target-level information fusion approach based on the MMW radar. Finally, we proposed an information-fusion-based fault diagnosis strategy centered on the Euclidean distance between the MMW radar and the camera centroid. We injected three separate failure modes into the camera sensors in turn; the simulation results indicate that for missing row/column pixel failure, the deviation typically falls between 34.11% and 99.84%, with a response time of 0.02 s to 1.6 s; for pixel shift faults, the deviation range is between 0.32% and 9.92%, with a response time of 0 s to 0.16 s; target color loss faults have a deviation range of 0.26% to 2.88%, and a response time of 0 s to 0.05 s. This demonstrates that the suggested method may identify sensor faults to achieve real-time fault alerts and ensure the safety and dependability of an autonomous driving system.

Further research can expand on this study in several ways. First, to enhance the robustness and efficiency of the system in practical applications, it is crucial to consider incorporating additional sensors besides MMW radar and cameras. The number of different sensors required can vary depending on the task and scenario and must be carefully evaluated. Second, in real-world scenarios, multiple sensors may simultaneously experience various types of faults, which necessitates the development of methods for detecting and isolating mixed sensor faults. This remains a challenging task for future research.

## Figures and Tables

**Table 1:** Failure modes and characteristics.

Failure Type	Failure Mode	Fault Characterization	Expected Test Results
No fault	—	—	τ<10% W
Failure fault	Missing row/column pixels	Missing pixels are white	τ≥30% W
Deviation fault	Pixel displacement	Blurred images	10% W≤τ<30% W
Deviation fault	Target color loss	Single image color	10% W≤τ<30% W

τ: the Euclidean distance between the MMW radar’s observation point and the camera’s center point, namely, the error between the center points of the two sensor tags. W: the target width of vehicle, W = 1.6 m.

**Table 2:** Motion parameter setting table.

Parameter	Value
Speed (m/s)	5.00
Roll friction coefficient	0.01
Drag coefficient	0.31
Mass (kg)	1471
Reference area (m^3^)	2.74
Air density (kg/m^2^)	1.28
Gravitation (m/s^2^)	9.81
Max acceleration (g)	0.30
Max deceleration (g)	0.30

**Table 3:** Sensor settings table.

Camera				MMW Radar			
Location	X	Y	Z	Location	X	Y	Z
(m)	2.000	0.000	1.320	(m)	3.940	0.000	0.370
Orientation	Bank	Pitch	Heading	Orientation	Bank	Pitch	Heading
(deg)	0.0	0.0	0.0	(deg)	0.0	0.0	0.0
Resolution	Horizontal		Vertical	Line scan direction		Left to Right/Top to Bottom	
(pixel)	320		240				
Frame rate (Hz)			20	Resulting scan frequency per beam (Hz)			20
Intensity factor (RGB)	0.30	0.59	0.11	Beam center line orientation (azimuth)(deg)		From −22.5 to 22.5	
—				Operating frequency (GHz)			25.000
				Max. objects to output			7

**Table 4:** Missing row/column pixel fault identification results.

	1	2	3	4	5	6	7	8	9	10
$\tau_{1}\left(m\right)$	0.92	1.39	0.75	1.57	0.10	0.05	0.16	1.43	1.01	0.24
$\tau_{1}/W\left(\%\right)$	57.25	86.64	47.03	97.87	6.05	3.21	10.22	89.21	62.84	15.30
$\tau_{2}\left(m\right)$	1.53	1.55	0.93	1.60	0.07	0.04	0.20	1.33	1.11	0.33
$\tau_{2}/W\left(\%\right)$	95.47	96.84	57.91	99.84	4.66	2.58	12.38	83.40	69.26	20.66
$\tau_{3}\left(m\right)$	1.53	1.27	0.55	1.52	0.02	0.05	0.13	0.83	1.43	0.29
$\tau_{3}/W\left(\%\right)$	95.47	79.53	34.11	95.23	1.23	2.93	7.99	52.17	89.22	18.42

**Table 5:** The outcomes of identifying pixel displacement faults.

	1	2	3	4	5	6	7	8	9	10
$\tau_{1}\left(m\right)$	0.04	0.10	0.13	0.01	0.16	0.14	0.13	0.08	0.01	0.10
$\tau_{1}/W\left(\%\right)$	2.77	6.07	8.10	0.32	9.92	9.02	8.39	5.13	0.63	5.97
$\tau_{2}\left(m\right)$	0.05	0.08	0.04	0.09	0.14	0.03	0.05	0.12	0.16	0.02
$\tau_{2}/W\left(\%\right)$	2.82	4.88	2.49	5.44	8.92	1.60	3.29	7.51	9.91	1.39
$\tau_{3}\left(m\right)$	0.09	0.02	0.02	0.04	0.12	0.10	0.00	0.09	0.12	0.01
$\tau_{3}/W\left(\%\right)$	5.34	1.27	1.40	2.43	7.28	6.42	0.02	5.87	7.47	0.83

**Table 6:** Target color loss fault identification results.

	1	2	3	4	5	6	7	8	9	10
$\tau_{1}\left(m\right)$	0.02	0.01	0.03	0.03	0.03	0.02	0.02	0.05	0.02	0.04
$\tau_{1}/W\left(\%\right)$	1.23	0.84	1.97	1.62	2.10	0.97	1.45	2.88	1.00	2.55
$\tau_{2}\left(m\right)$	0.03	0.02	0.04	0.01	0.04	0.04	0.00	0.01	0.02	0.01
$\tau_{2}/W\left(\%\right)$	1.77	1.35	2.33	0.71	2.43	2.48	0.26	0.58	1.19	0.37
$\tau_{3}\left(m\right)$	0.04	0.00	0.03	0.03	0.05	0.02	0.02	0.03	0.03	0.04
$\tau_{3}/W\left(\%\right)$	2.37	0.18	1.89	1.86	2.83	1.26	1.42	1.71	2.04	2.25

## Data Availability

The data presented in this study are available upon request from the corresponding author.
